# Neurokinin-1 receptor promotes non-small cell lung cancer progression through transactivation of EGFR

**DOI:** 10.1038/s41419-021-04485-y

**Published:** 2022-01-10

**Authors:** Xiao-Wei Zhang, Lin Li, Wen-Qian Hu, Ming-Ning Hu, Yan Tao, Hui Hu, Xiao-Kang Miao, Wen-Le Yang, Qiong Zhu, Ling-Yun Mou

**Affiliations:** 1 School of Life Science Lanzhou University, 222 TianShui South Road, Lanzhou, 730000 P. R. China; 2 Basic Medical Sciences & Research Unit of Peptide Science, Chinese Academy of Medical Sciences, 2019RU066, Lanzhou University, Lanzhou, 730000 P. R. China; 3 Key Laboratory of Preclinical Study for New Drugs of Gansu Province, School of Basic Medical Science, Lanzhou University, Lanzhou, 730000 P. R. China; 4 Key Laboratory of Urological Disease of Gansu Province, Lanzhou University Second Hospital, Lanzhou University, Lanzhou, 730000 P. R. China

**Keywords:** Non-small-cell lung cancer, Mechanisms of disease

## Abstract

Despite the great advances in target therapy, lung cancer remains the top cause of cancer-related death worldwide. G protein-coupled receptor neurokinin-1 (NK1R) is shown to play multiple roles in various cancers; however, the pathological roles and clinical implication in lung cancer are unclarified. Here we identified NK1R as a significantly upregulated GPCR in the transcriptome and tissue array of human lung cancer samples, associated with advanced clinical stages and poor prognosis. Notably, NK1R is co-expressed with epidermal growth factor receptor (EGFR) in NSCLC patients’ tissues and co-localized in the tumor cells. NK1R can crosstalk with EGFR by interacting with EGFR, transactivating EGFR phosphorylation and regulating the intracellular signaling of ERK1/2 and Akt. Activation of NK1R promotes the proliferation, colony formation, EMT, MMP2/14 expression, and migration of lung cancer cells. The inhibition of NK1R by selective antagonist aprepitant repressed cell proliferation and migration in vitro. Knockdown of NK1R significantly slowed down the tumor growth in nude mice. The sensitivity of lung cancer cells to gefitinib/osimertinib is highly increased in the presence of the selective NK1R antagonist aprepitant. Our data suggest that NK1R plays an important role in lung cancer development through EGFR signaling and the crosstalk between NK1R and EGFR may provide a potential therapeutic target for lung cancer treatment.

## Introduction

Lung cancer remains the top cause of cancer-related death in China and worldwide for years and non-small cell lung cancer (NSCLC) accounts for more than 80% of lung cancer cases [1, 2]. Tyrosine kinase inhibitors of EGF receptor (EGFR TKI) have dramatically changed the therapeutic paradigm of NSCLC [3], which are generated to bind and inhibit the intracellular kinase domain of EGFR with positive EGFR mutations [4]. Molecularly selected NSCLC patients with point mutation L858R or in-frame deletion of exon 19 (Ex19del) in EGFR (10–40% of NSCLC cases depending on the ethnicity of the population) are highly responsive to the first- and second-generation EGFR TKIs like gefitinib and apatinib [5, 6]. However, the development of acquired resistance was almost inevitable and secondary EGFR mutations, commonly T790M, occurred in about half of the relapsed patients. Osimertinib, the third-generation EGFR TKI was approved to overcome the resistance of T790M mutation [7]; however, acquired resistance has already been detected in clinical treatment [8, 9]. Notably, all approved EGFR TKIs are gene-type specific and much less efficient in patients with wtEGFR amplification or other insensitive mutant EGFRs (such as Ex20ins); thus, there is still a large part of NSCLC patients who cannot benefit from current EGFR-targeting therapeutic strategies. The failures highlight the unmet need to identify other targets serving to promote tumor progression, which may be developed into novel strategies for NSCLC therapy.

GPCR is the largest superfamily of cell membrane proteins, often be linked with pathological situations and translated into clinical therapeutic targets [10]. Recent studies have recognized an essential role of GPCR in tumor initiation and development [11], although there remains a plethora of knowledge in this area yet to be understood. Indeed, several peptide mimics targeting GnRH receptor and somatostatin receptor, and vismodegib, a small-molecule inhibitor of the smoothened receptor have been approved for the treatment of various types of cancer [12, 13]. GPCRs are usually considered to exert biological functions by coupling with heterotrimeric G proteins and mediating downstream signaling pathways. However, a growing number of evidence show that ligand activation of GPCR can also induce transactivation of EGFR in various cancer cells, which represent an important mechanism of regulating both GPCR- and EGFR-mediated tumor progression [14–18]. Activated GPCR can upregulate matrix metalloproteases (MMPs) level that release the membrane-bound EGFR ligands such as heparin-binding EGF-like factor (HB-EGF) and TGFα, then activating EGFR and downstream signaling pathways [19, 20]. Except the ligand-dependent way, increasing studies indicate that adaptor protein like c-Src and β-arrestin may be involved with the transactivation of EGFR by GPCR [21, 22]. Actually, the crosstalk between GPCRs and EGFR has been shown to make contributions to the initiation and progression of colon, breast, ovarian, prostate, and head and neck tumor [23]. In NSCLC tumor cells, several GPCRs have been identified to be overexpressed such as gastrin-releasing peptide, pituitary adenylate cyclase-activating polypeptide, and bombesin receptor. They played roles in mediating NSCLC cell proliferation, metastasis, and drug resistance by transactivating EGFR signaling [15, 24, 25], implying that the crosstalk between GPCR and EGFR may provide the opportunities to discover novel pharmacological approaches to treat lung cancer patients.

Neurokinin-1 receptor (NK1R) is a GPCR belonging to tachykinin receptor family. Besides the physiological roles in emotion, vomit reflex, pain transmission, and inflammation, accumulating studies indicate NK1R as an oncogenic GPCR [26]. NK1R was found to be upregulated in tumor tissues such as melanoma, gliomas, and breast cancer [27–29]. Its endogenous ligands, peptide substance P and hemokinin-1 (HK-1), exhibit the tumorigenesis ability and facilitate tumor cells to resist chemotherapy-induced apoptosis by activating the receptor [30–32]. The potential crosstalk of EGFR and NK1R has been described in breast cancer and glioblastoma, showing that NK1R activation affected EGFR phosphorylation and downstream signaling pathways, thus regulating cancer cell proliferation and drug sensitivity. In lung cancer, several studies reported that SP could promote cell proliferation, while the NK1R antagonists exerted antiproliferative effect and promoted cell apoptosis [33–35]. These data suggested NK1R as a pro-tumorigenic GPCR in NSCLC; nevertheless, the detailed molecular mechanisms remain largely unknown. In this study, we found that NK1R expression is significantly increased in the tumor tissues of NSCLC patients. The upregulation of NK1R is positively related to the EGFR expression level, signaling activity and NSCLC progression. The crosstalk between NK1R and EGFR is involved with the protein–protein interaction of the two receptors. Inhibition of NK1R increased the sensitivity of NSCLC cells to gefitinib and osimertinib. Our findings suggest that NK1R plays an important role through transactivating EGFR in NSCLC progression and highlights its potential as a target for NSCLC therapy.

## Materials and methods

### Cell culture and reagents

Human NSCLC cell lines NCI-H1975, NCI-H1944, NCI-H226, A549, HCC827 were purchased from American Type culture collection (ATCC, Manassas, VA, USA) and cultured in RPMI-1640 medium supplemented with 10% fetal bovine serum (ThermoFisher, Waltham, USA) and 1% penicillin/streptomycin solution (Sangon, Shanghai, China). The cells were cultured in a humidified incubator at 37 °C with 5% CO_2_. EGFR genotyping was performed in each cell line according to the instruction of the manufacture (Yihe, Shanghai, China). Aprepitant was obtained from MedChemExpress (NJ, USA). Osimertinib and gefitinib were from Selleck Chemicals (Houston, TX, USA). All chemicals were dissolved in dimethyl sulfoxide at the concentration of 100 mM and aliquoted and stored at −20 °C until use. Human hemokinin-1 (hHK-1) peptide (TGKASQFFGLM-NH_2_) and substance P (SP) (RPKPQQFFGLM-NH2) were synthesized by Fmoc solid-phase synthesis system as described before [28]. The molecular weight of the peptide was confirmed by ESI-TOF mass spectrometry. The purity of peptide was quantified to be >95% using reversed-phase HPLC by a C18 column as the solid phase and a H_2_O:acetonitrile gradient as the liquid phase.

### Tissue microarray

A tissue array containing 30 pairs of human lung adenocarcinomas and matched adjacent non-tumor tissues was purchased from Superchip Biotechnology (Shanghai, China). The tissue microarray contained 3 paired I–II grade tissues, 11 paired II grade tissues, 13 paired II–III grade tissues, and 3 paired III grade tissues. Other information about the lung cancer samples is shown in Supplementary Table 1.

### Transfections and plasmid

Lentivirus was obtained from OBIO Technology (Shanghai, China). The sequence of shRNA (shNK1R 1#: 5′-GCAACCAGCCTGGCAAATT-3′; shRNA NK1R 2#: 5′-GCCTGTTCTACTGCAAGTT-3′; shctrl: 5′-TTCTCCGAACGTGTCACGT-3′) were designed, synthesized, and cloned into the lentiviral vector pLKD-CMV-EGFP-Puro. Cells were infected with lentivirus particles containing shRNA NK1R 1#, 2# or control according to the manufacturer’s instructions. For NK1R overexpression, cells were transfected with pLenti-EGFP-Puro-CMV-NK1R vector (OBIO) by lipofectamine2000 (ThermoFisher). The efficiency of NK1R knockdown and overexpression was detected by western blot and real-time quantitative PCR following puromycin selection (5 μg/ml, Solarbio, Beijing, China).

For EGFR knockdown, we transfected cells with dual sgRNA Crispr-Cas9 system Lenti-CRISPR- V2 dual sgRNA plasmid according to the instruction of the manufacture (Genecarer, Xi’an, China). The target sgRNA sequence was the followed: sgRNA1 (F: 5′-GCCTTAATACCTGGACCTTGA-3′; R: 5′-GTCCAGACTCTTTCGATACCC-3′); sgRNA2 (F: 5′-GAGCTTTGCGCCCAGATGACC-3′; R: 5′-GTCTAAGAGCTAATGCGGGCA-3′). EGFR expression was detected by western blot.

### mRNA purification and real-time quantitative PCR

Total RNA was extracted by RNAiso Plus according to the instruction of the manufacture (Takara, Dalian, China). One microgram of purified RNA dissolved in DNase/RNase-free water was reversely transcribed into cDNA using PrimeScript^TM^ RT master mix reagents (Takara). Real-time quantitative PCR reactions were performed by TB Green Premix ExTaq kit (Takara) in Applied Biosystems QuantStudio instrument (ThermoFisher). The real-time quantitative PCR condition was as follows: 30 s at 95 °C, 40 cycles of 5 s at 95 °C, followed by 20 s at 60 °C. All primers sequences are synthesized by Sangon (Shanghai, China) and listed in Supplementary Table 2. The relative expression of genes was normalized by β-actin. Data were analyzed and shown as −ΔΔCT.

### Western blot analysis

Cells were plated and cultured overnight, then incubated with chemicals as indicated. Total protein of whole cell lysate was prepared by RIPA lysis buffer (Beyotime, Shanghai, China) with 1 mM phenylmethylsulfonyl fluoride (Sangon) and Phosphatase inhibitor cocktail (Beyotime). The protein concentration of cell lysate was determined by BCA kit (ThermoFisher). An equal amount of 20~30 μg protein sample was loaded and separated by 10% SDS-PAGE, and then transferred to PVDF membranes (Millipore, MA, USA). After blocking in 6% skimmed milk containing 0.05% tween-20 (Sangon) for 2 h at room temperature, the membranes were incubated with primary antibodies (1:1000) at 4 °C overnight and subsequently with HRP-labeled secondary antibodies (1:2000) for 2 h at room temperature. The protein bands were detected by Enhanced Chemiluminescence kit (New Cell & Molecular Biotech, Suzhou, China) and visualized in Chemiluminescence Imaging System (Cytiva, Japan). The relative band intensity of each blot was analyzed by Image J software and normalized to GAPDH. The primary antibodies anti-pEGFR (Try1068) (#3777), anti-EGFR (#2085), anti-pERK1/2 (#4370), anti-pAKT (#4060), anti-E-cadherin (#3195), and anti-Vimentin (#5741) were from Cell Signaling Technology (Danvers, MA, USA); anti-ERK (AF1051) and anti-AKT (AA326) were from Beyotime; anti-GAPDH (10494-1-AP) was from ProteinTech Group (Wuhan, China); anti-NK1R (ab183713), anti-MMP2 (ab92536), and anti-MMP14 (ab51074) were from Abcam (Cambridge, UK); anti-Bcl-2 (sc-7382) and anti-Bax (sc-7480) were from Santa Cruz (CA, USA). The HRP-labeled secondary antibody goat anti-rabbit IgG (H + L) and goat anti-mouse IgG (H + L) were from Beyotime. The other detailed information of the antibodies and reagents is listed in Supplementary Table 3.

### Co-immunoprecipitation

Co-immunoprecipitation was performed to detect the protein–protein interaction between NK1R and EGFR as described before [36]. Total protein was extracted from 1 × 10^6^ cell cultures using RIPA lysis buffer with PMSF and Phosphatase inhibitor cocktail. Eighty microgram protein sample was immunoprecipitated with anti-EGFR (CST, #2085), anti-NK1R (Abcam ab183713), or IgG (CST, #7074) diluted at 1:100 at 4 °C overnight. The immunoprecipitated protein was incubated with 40 μl protein A+G agarose beads (Beyotime) at 4 °C for 3 h, and then washed with PBS three times. The immunoprecipitated protein was immunoblotted by corresponding primary antibodies and visualized in Chemiluminescence Imaging System.

### Flow cytometry

NK1R expression on the cell surface was detected by flow cytometry as described before [37]. A total of 1 × 10^6^ cells were harvested and stained with anti-NK1R (Abcam, ab183713) for 30 min at room temperature, followed by incubation with coralite594-conjugated secondary antibody (ProteinTech) for 30 min at room temperature. Then the cells were washed with PBS three times before flow cytometry analysis. For cell apoptosis analysis, 1 × 10^6^ cells were treated with aprepitant for 24 h. Then cells were fixed with 75% ethanol, washed by cold PBS three times before incubating with Annexin V-FITC/PI (Yuheng Biotechnology, Suzhou, China) according to the manufacturer’s instructions. Data were acquired by BD LSRFortessa Flow Cytometer (BD Biosciences, Franklin Lake, NJ, USA) and analyzed using the FlowJo software (BD Biosciences, USA).

### Confocal microscopy analysis

A total of 1 × 10^4^ cells were plated in a glass bottom/confocal petri dish (Biosharp, Hefei, China) and cultured overnight. Cells were fixed by 4% paraformaldehyde solution (Beyotime) for 10 min and blocked with immuno-staining blocking buffer (Beyotime) for 60 min at room temperature, then incubated with mouse anti-EGFR (ProteinTech, 66455-1-lg) and rabbit anti-NK1R (Abcam, ab183713) overnight at 4 °C. The cells were then stained with coralite594-conjugated anti-rabbit IgG (ProteinTech, SA0013-4) and coralite488-conjugated anti-mouse IgG (ProteinTech, SA0013-1) for 2 h at room temperature. Nuclei were stained by DAPI (1 μg/ml, Sangon). Fluorescent images were captured by a microscope equipped with a laser-scanning confocal imaging system (Zeiss LSM800, German).

### Tumor xenografts models

All animal experiment procedures were approved by the Ethics Committee of Animal Experimentation and performed following the institutional guidelines of animal care (Center of Medical Experiment, Lanzhou University, China). Male athymic nude BALB/C mice of 4–5 weeks old were purchased from GemPharmtechnology (Jiangsu, China), weighing 16–18 g, and kept under specific pathogen-free conditions in the animal room. A total of 3 × 10^6^ cells transfected with shRNA NK1R 2# or control shRNA were suspended in 100 μl mixture of Matrigel matrix (Corning, New York, USA) and injected into the right flank of nude mice. Tumor volume was measured every 2 days after cells injection. The tumor size was calculated according to the following formula: tumor volume = 1/2 × length (mm) × width (mm)^2^. At indicated time point, mice were euthanized, and tumor tissues were isolated. The tumor tissues were fixed in 4% formalin and embedded in paraffin for tissue sections.

### Immunohistochemistry (IHC)

IHC experiment was conducted to examine the expression of NK1R, EGFR, Ki67, pERK1/2, and pAkt in human lung cancer tissue microarray and/or tumor tissue sections from the xenograft NSCLC models. The slices were dewaxed and rehydrated by a standard xylene-ethanol procedure. The slides were heated in 10 mM sodium citrate buffer (pH 6.0) for 2 min to repair the antigen. Endogenous peroxidase was blocked by 3% H_2_O_2_ for 10 min at room temperature. Non-specific sites were blocked by normal goat serum for 18 min after being washed with PBS. Finally, slides were incubated with corresponding primary antibodies at 4 °C overnight. An appropriate HRP-labeled secondary antibody was incubated with the slides at room temperature for 30 min (ZSGB-BIO, Beijing, China). DAB (Service, Wuhan, China) was added to visualize the protein expression with cell nuclei gently counterstained with hematoxylin (Beyotime). Tissue sections were stained with hematoxylin and eosin for histomorphometric analysis. The results were captured by NIS-element imaging system (Nikon, Japan).

### Cell viability and colony formation assay

Cell viability was evaluated by CellTiter-Glo luminescence assay according to the manufacturer instruction (Promega, Madison, WI, USA). To detect the effect hHK-1 on cell growth, 1 × 10^3^ NSCLC cells were seeded and cultured in 96-well plates with hHK-1 at indicated time course. To detect the effect of aprepitant and/or gefitinib/osimertinib on cell growth, 5 × 10^3^ cells were seeded in 96-well culture plate and treated with chemicals as indicated for 72 h. The luminescence was read by Flex Station III microplate reader (Molecular Devices, Sunnyvale, Silicon Valley, USA). Each experiment was performed at least three times with three repeats. For colony formation assay, 500 cells of each NSCLC cell line were seeded into 6-well plate and dealt with indicated drugs. Every 3 days the cells were replaced with a fresh culture medium containing indicated drugs for 2 weeks. Cells were fixed by 90% ethanol and stained with 0.2% crystal violet (Sangon). The plates were scanned and pictured by a camera. Colonies >1 mm were counted by Image J.

### Transwell migration assay

Cells suspended in 100 μl 1640 medium with 2% FBS were seeded into the upper chamber of transwell chamber (Corning), then inserted in a 24-well plate containing 600 μl 1640 medium with 2% FBS and the indicated concentration drugs for incubation of 24 h in 37 °C with 5% CO_2_. The migrated cells were fixed by 90% ethanol for 30 min and stained with 0.2% crystal violet for 20 min at room temperature after being washed by PBS. Non-migrated cells were removed by cotton swabs, and migrated cells were washed by PBS three times. After air-drying, cell images were captured and counted in three different random areas by microscope with an image processing system (Nikon, Japan).

### Bioinformatics analysis

Gene expression profiling of lung cancer data GSE130779 and GSE2514 was acquired from public database Gene Expression Omnibus (GEO). GSE130779 composed of eight adjacent non-tumor tissues and eight adenocarcinoma tissues. GSE2514 contained 19 normal tissues and 20 tumor tissues. NK1R and EGFR expression levels were analyzed in GSE130779 and GSE2514 datasets by GraphPad Prism software. The Kaplan–Meier survival analysis was performed on Kaplan–Meier Plotter website (http://kmplot.com/analysis/index.php?p=service&cancer=lung) in datasets from GEO and The Cancer Genome Atlas (TCGA) (CAARRAY, *n* = 504; GSE14814, *n* = 90; GSE19188, *n* = 156; GSE2913, *n* = 55; GSE30219, *n* = 307; GSE31210, *n* = 246; GSE3141, *n* = 111; GSE31908, *n* = 40; GSE37745, *n* = 196; GSE43580, *n* = 150; GSE4573, *n* = 130; GSE50081, *n* = 181; GSE8894, *n* = 138; TCGA, *n* = 133).

### Statistical analysis

Data were presented as mean ± standard deviation (SD) calculated by GraphPad Prism 7.00 software (Chicago, USA). Statistical significance between different groups was calculated by one-way ANOVA with Tukey’s test and two-tailed Student’s *t*-test. Pearson analysis was performed to show the correlation between NK1R and EGFR expression. Log-rank test was used to Kaplan–Meier analysis. It was identified as statistically significant when *P* value <0.05 (*P < 0.05, **P < 0.01, ***P < 0.001).

## Results

### Upregulation of NK1R is associated with NSCLC development and poor prognosis

**Fig. 1:**
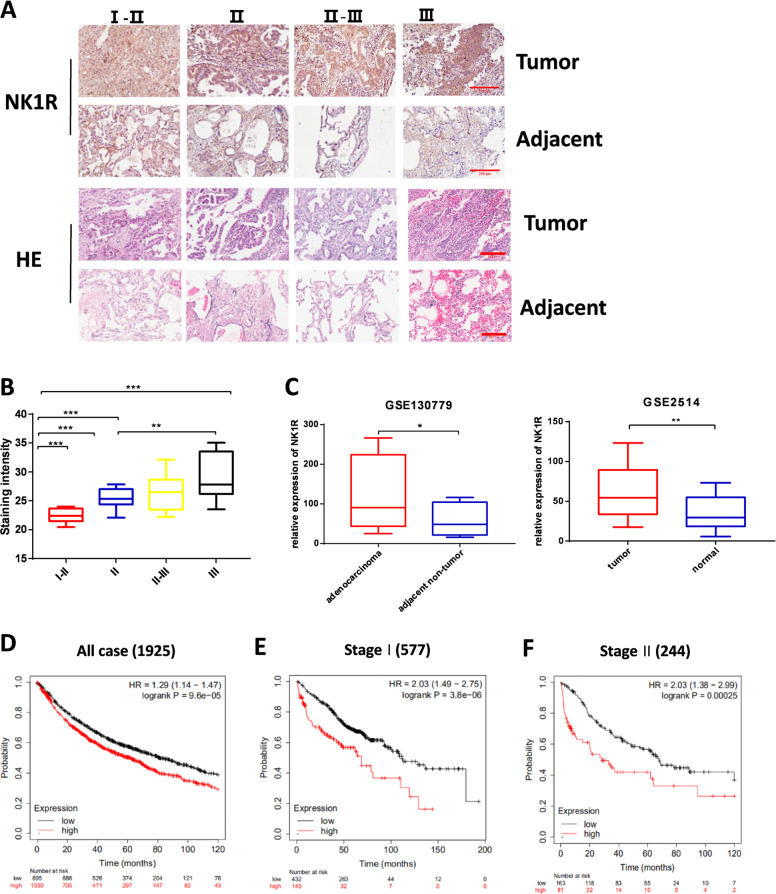
Expression level of NK1R is related to NSCLC progression and poor prognosis. IHC staining of NK1R was performed on tissue microarrays of 30 human lung adenocarcinoma patients. **A** The IHC analysis of NK1R expression in tumor tissues compared with matched adjacent tissues from stage I–II (*n* = 3), stage II (*n* = 11), stage II–III (*n* = 13), to stage III (*n* = 3). Scale bar, 200 μm. **B** IHC staining intensity of NK1R in tumor tissues of lung adenocarcinoma from stage I to stage III. **C** NK1R expression analysis in human lung cancer in comparison with the matched adjacent tissues in GSE130779 and with normal tissues in GSE2514. **D**–**F** The Kaplan–Meier overall survival analysis with the log-rank test in 1925 cases from GEO and TCGA datasets (see details in Methods), defined by tumor stages and by low/high NK1R expression. *P < 0.05, **P < 0.01, ***P < 0.001.

To investigate its potential role in NSCLC, the expression of NK1R was detected in a tissue array consisting of 30 samples of human lung adenocarcinoma. IHC staining showed that NK1R expression level was significantly higher in tumor tissues than the matched adjacent normal tissues. IHC staining intensity analysis suggested that NK1R level was positively correlated with the clinical development stage of NSCLC (Fig. 1A, B and Supplementary Fig. 1). We further analyzed NK1R expression in NSCLC and adjacent non-tumor tissues in two datasets GSE130779 and GSE2514 downloaded from GEO databases [38, 39], revealing that expression of NK1R was significantly upregulated in NSCLC tumor tissues compared with the adjacent non-tumor tissues or normal tissues (Fig. 1C). The relationship between the expression level of NK1R and NSCLC prognosis was evaluated by Kaplan–Meier survival analysis (http://kmplot.com/analysis/index.php?p=service&cancer=lung). A total of 1925 lung cancer patient cases from GEO and TGCA dataset were divided into two groups by best cutoff value of NK1R expression: low NK1R (*n* = 895), high NK1R (*n* = 1030). High NK1R (NK1R^high^) group was associated with poor prognosis, accompanied by the median survival time of 62 months (95% CI: 43–82 months). In contrast, the low NK1R (NK1R^low^) group showed a better prognosis with the median survival time of 81.1 months (95% CI: 76–121 months) (Fig. 1D). Significantly, patients with lower NK1R showed a higher overall survival rate in both stage I and stage II groups (Fig. 1E, F). Together, these results suggested that NK1R expression was significantly upregulated in NSCLC patients and there was a potent relevance between NK1R expression level and the progression of NSCLC along with a poor prognosis.

### NK1R promotes NSCLC progression in vitro

**Fig. 2:**
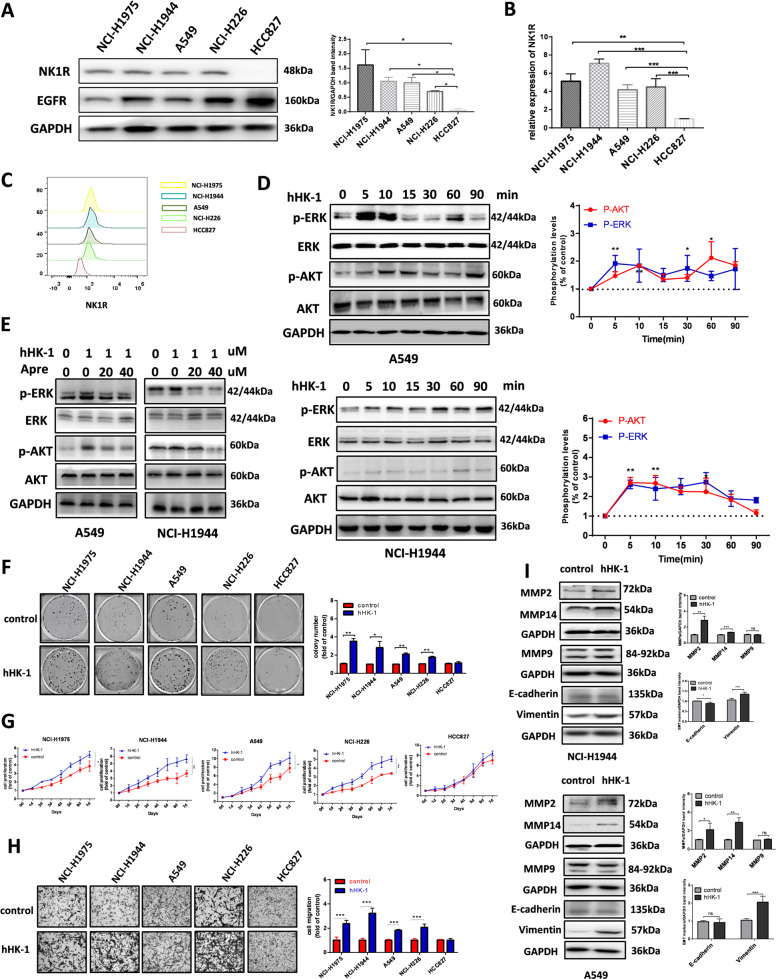
NK1R promotes NSCLC progression in vitro. **A** Western blot and **B** real-time PCR analysis of NK1R expression in NSCLC cell lines. **C** FACS analysis of NK1R expression on cellular surface membrane. HCC827 cell lysate was used as a negative control. **D** Western blot analysis of hHK-1 induced ERK1/2 and Akt phosphorylation in NSCLC cell lines. **E** Aprepitant reduced the phosphorylation of ERK1/2 and AKT triggered by hHK-1. Total ERK1/2 and Akt were used as a loading control. The effect of NK1R activation by 1 μM hHK-1 on **F** cell colony formation (colonies larger than 0.1 mm in diameter were counted), **G** cell growth curve, **H** transwell cell migration, and **I** E-cadherin/vimentin, MMP2/14 protein level. The densitometric analysis of each band was calculated with Image J software. Data were shown as mean ± SD from three independent experiments. *P < 0.05, **P < 0.01, ***P < 0.001.

Western blot and real-time quantitative PCR were performed to examine the expression level of NK1R in NSCLC cell lines. NK1R was highly expressed in NSCLC cell line NCI-H1944, NCI-H1975, NCI-H226, A549, and relatively lower in HCC827 (Fig. 2A, B). In consistence, the result of FACS analysis indicated that NK1R was obviously detected on cell membrane of NCI-H1944, NCI-H1975, NCI-H226, A549, and relatively lower in HCC827 (Fig. 2C). To determine whether NK1R functioned in NSCLC cells, we evaluated the influence of NK1R on intracellular signaling in different NSCLC cells. hHK-1 is an endogenous peptide agonist of NK1R [8, 10]. It was observed that hHK-1 induced significant phosphorylation of ERK1/2 and Akt in a time-dependent way while the selective antagonist of NK1R aprepitant inhibited the effect dose-dependently (Fig. 2D, E and Supplementary Fig. 2). NK1R-mediated MAPK and Akt signaling pathways have been involved with tumor cell proliferation and migration. In NK1R^high^ cell lines (referring to NCI-H1944, NCI-H1975, NCI-H226, and A549), colony formation assay results described an enhanced cell proliferative ability in hHK-1-treated group (less efficient in NK1R^low^ HCC827) (Fig. 2F). The growth curve of the cell lines showed that hHK-1 accelerated the cell growth in all the NK1R^high^ NSCLC cell lines but not in NK1R^low^ HCC827 (Fig. 2G). hHK-1 administration also remarkably elevated the migration ability in NK1R high NSCLC cells indicated by the increased invading cell number (Fig. 2H). To further confirm, we used a second NK1R ligand SP, which showed a similar effect to activate receptor-mediated signaling pathway as well as stimulating cell proliferation and migration (Supplementary Fig. 3A–C).

Epithelial-mesenchymal transition (EMT) is key step of tumor metastasis characterized by the expression level of epithelial marker E-cadherin and mesenchymal marker Vimentin. With hHK-1 treatment for 24 h, vimentin remarkably increased in NK1R^high^ cell lines but not in HCC827 cells. In addition, hHK-1 augmented the protein level of MMP2 and −14 (Fig. 2I and Supplementary Fig. S4). Collectively, these results showed that the activation of NK1R promoted the progression of NSCLC cells in vitro.

### Inhibition of NK1R suppresses NSCLC cell progression in vitro

**Fig. 3:**
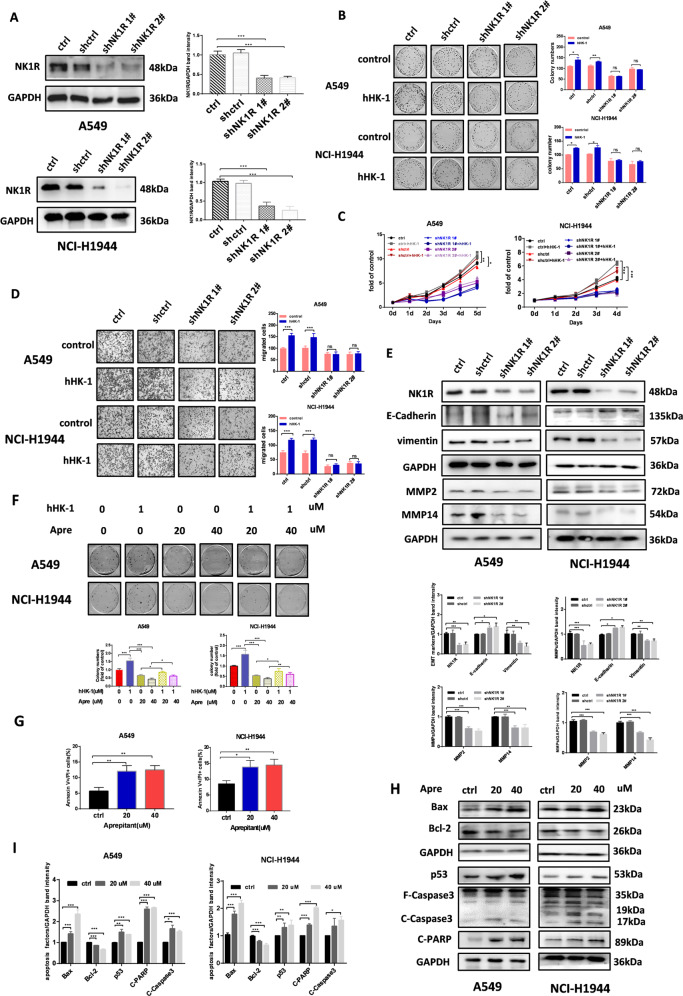
Inhibition of NK1R suppresses NSCLC cell progression in vitro. **A** Western blot analysis of NK1R expression in NSCLC cells transiently transfected with shRNA targeting NK1R or control shRNA. The effect of shRNA-mediated NK1R knockdown on **B** NSCLC cell colony formation (colonies larger than 0.1 mm in diameter were counted), **C** cell growth curve, **D** transwell cell migration, and **E** E-cadherin/vimentin, MMP2/14 protein expression. **F** Aprepitant inhibited the ability of cell colony formation stimulated by hHK-1. Aprepitant treatment induced NSCLC cell apoptosis measured by **G** flow cytometry analysis of PI/Annexin V co-staining and **H** western blot analysis of p53, Bax, Bcl-2, caspase3, cleaved-PARP protein level. **I** Band intensity of the p53, Bax, Bcl-2, caspase3, cleaved-PARP protein. migrated cell number, and densitometric analysis of each band were calculated with Image J software. Data were shown as mean ± SD from three independent experiments. *P < 0.05, **P < 0.01, ***P < 0.001.

We transfected NCI-H1944 and A549 cells with specific shRNA targeting NK1R (Fig. 3A). The reduction of NK1R expression distinctively inhibited the colony formation ability (Fig. 3B). Cell growth curve showed that the proliferation of both cell lines was attenuated compared with un-transfected and control-shRNA groups (Fig. 3C). The migrative cell number decreased in shNK1R-transfected cells, along with the reduced protein level of vimentin, MMP2 and −14, showing the reduced ability of tumor cell metastasis (Fig. 3D, E). In addition, shNK1R-transfected A549 and NCI-H1944 cells showed no response to hHK-1 treatment. Aprepitant reduced cell proliferation and blocked hHK-1-induced cell colony formation (Fig. 3F). Flow cytometry of PI/Annexin V co-staining assay showed that aprepitant induced cell apoptosis dose-dependently (Fig. 3G). Western blot assay showed that aprepitant significantly increased p53 expression, enhancing the protein level of pro-apoptotic factor Bax while reducing the level of anti-apoptotic Bcl-2, increasing the level of cleaved caspase3 and PARP (Fig. 3H, I). Taken together, these results suggested that inhibition of NK1R suppressed NSCLC tumor progression in vitro and that the treatment of NK1R antagonist promoted NSCLC tumor cell apoptosis.

### Knockdown of NK1R impaired NSCLC tumor growth in vivo

**Fig. 4:**
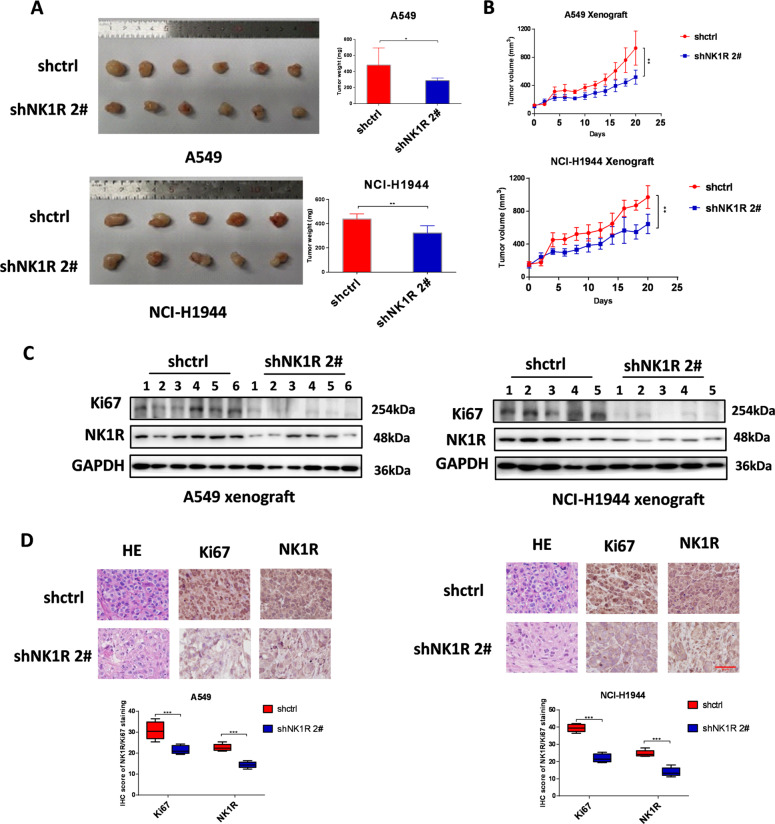
Knockdown of NK1R impaired NSCLC tumor growth in vivo. A549 and NCI-H1944 cells were transfected with control or shNK1R plasmid. **A**, **B** The effect of NK1R knockdown on NSCLC tumor growth in nude mice. **C** Western blot and **D** IHC analysis of NK1R and Ki67 protein level in xenograft tumor samples. Scar bar, 50 μm. The IHC staining and densitometric analysis of each band were calculated with Image J software. Data were shown as mean ± SD from three independent experiments. *P < 0.05, **P < 0.01, ***P < 0.001.

To investigate the influence of NK1R expression on NSCLC tumorigenesis in vivo, we established the xenograft model derived from NSCLC cells. NCI-H1944 and A549 cells were transfected with NK1R-specific shRNA and injected into the right flank of nude mice. As shown in Fig. 4A, B, in comparison with the control group, tumor tissues initiated by NK1-knockdown NSCLC cells had significantly slower growth with smaller tumor sizes and lower tumor weights. In addition, IHC assay and western blot exhibited that NSCLC tissues from cells with knockdown NK1R had significantly decreased proliferative index of Ki67 compared with the control group (Fig. 4C, D). Furthermore, the level of phosphorylated ERK1/2 and Akt was significantly reduced in NK1R-knockdown tumor tissues (Supplementary Fig. 5). These results suggested an oncogenic role of NK1R in NSCLC tumor development in vivo.

### NK1R expression was correlated with EGFR in NSCLC

**Fig. 5:**
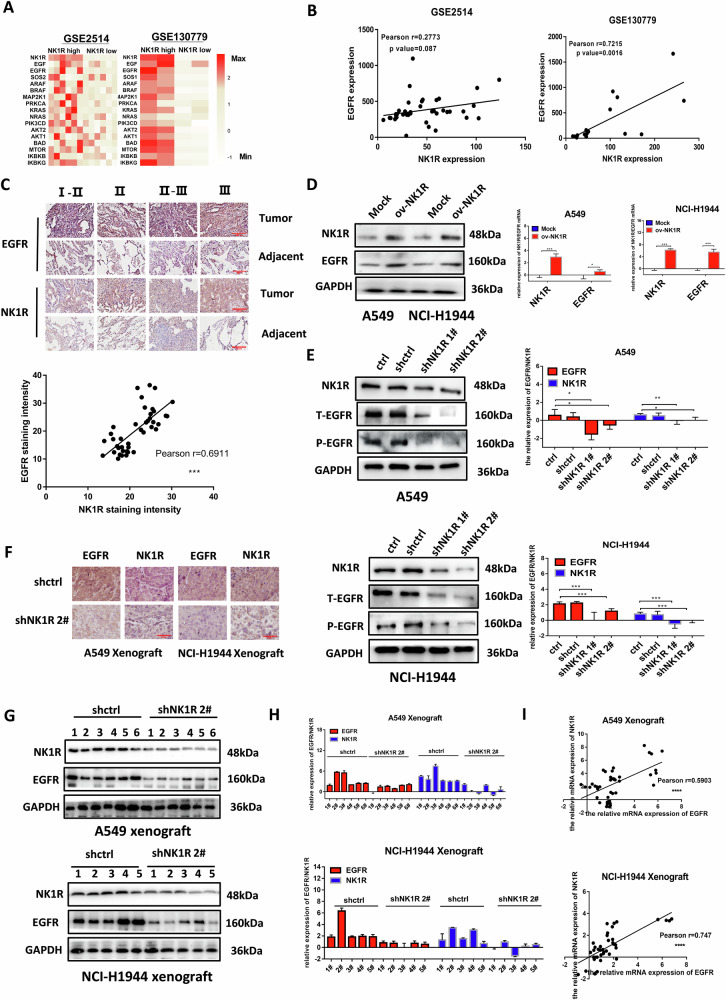
NK1R expression was correlated with EGFR in NSCLC. **A** Heatmap showing the differential NK1R expression and EGFR signaling activity in GSE2514 (NK1R^high^, *n* = 6; NK1R^low^, *n* = 6) and GSE130779 (NK1R^high^, *n* = 2; NK1R^low^, *n* = 2). **B** mRNA expression level of NK1R was plotted against EGFR in patient samples from GSE2514 and GSE130779. **C** IHC staining of NK1R and EGFR protein expression in tissue microarrays of 30 human lung adenocarcinoma patients (upper panel). IHC staining intensity of NK1R was plotted against EGFR (down panel). Scale bar, 200 μm. **D** Western blot and **E** real-time PCR analysis of NK1R and EGFR expression in NSCLC cells transfected by pLenti-EGFP-Puro-CMV-NK1R plasmid (NK1R overexpression, ov-NK1R) or by shRNA targeting NK1R (shNK1R). **F** IHC staining (scale bar, 50 μm), **G** Western blot and **H** real-time PCR analysis of NK1R and EGFR protein expression in the tissue samples of nude mice xenograft models. **I** mRNA expression level of NK1R was plotted against EGFR in tissue samples of nude mice xenograft models. The IHC staining and densitometric analysis of each band were calculated with Image J software. Data were shown as mean ± SD from three independent experiments. *P < 0.05, **P < 0.01, ***P < 0.001.

By analyzing NK1R mRNA expression and EGFR signaling from published NSCLC patient profiles (GSE130779 and GSE2514) [38, 39], we found that high NK1R expression was associated with EGFR signaling activity (Fig. 5A), implying that NK1R upregulation may be associated with enhanced EGFR function in NSCLC. In two analyzed GEO datasets, the mRNA level of NK1R was positively related to EGFR (Fig. 5B). Moreover, an NSCLC tissue array consisting of 30 human lung adenocarcinoma samples was evaluated by IHC staining, demonstrating that NK1R protein level was positively related to EGFR expression (Fig. 5C). Since the correlation of NK1R and EGFR was implicated at both mRNA and protein level, we overexpressed NK1R in NCI-H1944 and A549 cells. Western blot and real-time quantitative PCR results revealed that the NK1R-ov cells showed not only an elevated NK1R expression level but also an enhanced level of EGFR (Fig. 5D); in contrast, shRNA transfection mediated NK1R knockdown along with reduced protein level of EGFR (Fig. 5E). IHC staining of NSCLC tissue samples from NSCLC cell-initiated xenograft models also indicated that the expression of EGFR was downregulated in NK1R-knockdown group (Fig. 5F). In consistence, real-time quantitative PCR and western blot confirmed the reduction of EGFR protein and mRNA level in the xenograft-derived tissue samples (Fig. 5G–I). Taken together, these results suggested that NK1R expression was positively correlated with EGFR expression in NSCLC.

### NK1R was able to interact with EGFR and transactivate EGFR in NSCLC cells

**Fig. 6:**
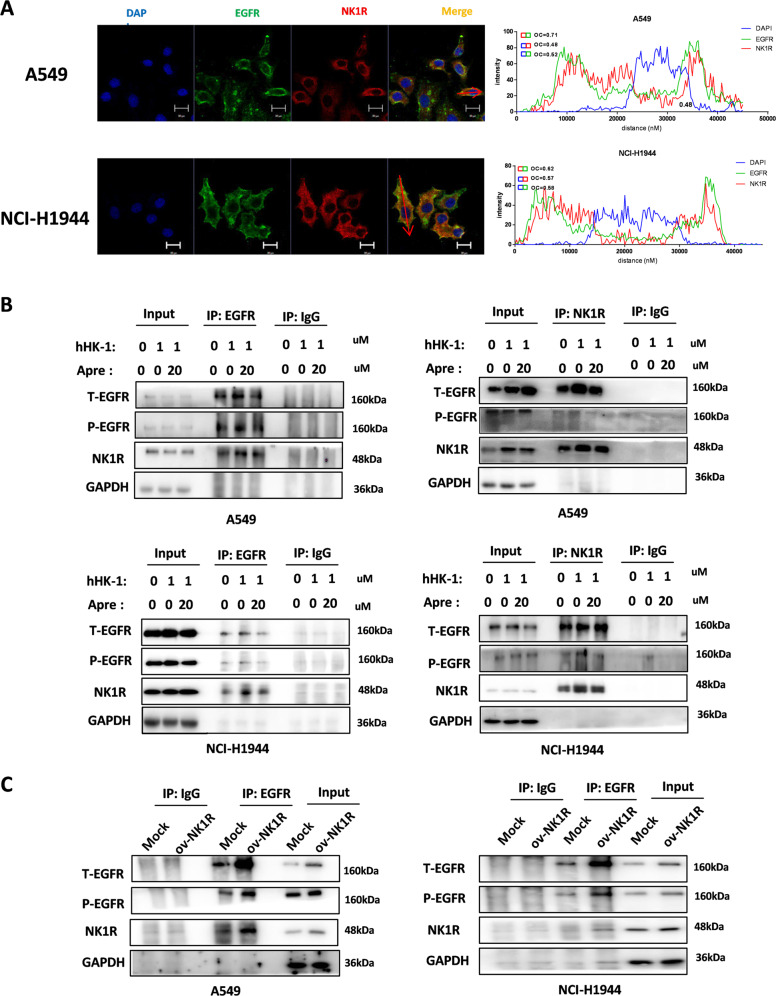
NK1R interacts with EGFR in NSCLC cells. **A** Confocal microscopy analysis to detect the colocalization of EGFR and NK1R in A549 and NCI-H1944. Green, EGFR; Red, NK1R; nucleus was stained by DAPI. Scale bar, 20 μm. The plot profile of fluorescence intensity was used to show the overlap of the NK1R and EGFR. Overlap Coefficient (OC) was >0.6 as the indication colocalization, while overlap coefficient was <0.6 as the absence of colocalization. **B** Co-immunoprecipitation assay by the antibody of NK1R or EGFR to show NK1R-EGFR interaction in A549 and NCI-H1944 cell lines. Cells were treated with hHK-1 or aprepitant for 24 h to detect the effect on NK1R-EGFR interaction. **C** Overexpression of NK1R in A549 and NCI-H1944 enhanced NK1R-EGFR interaction in a co-immunoprecipitation assay.

**Fig. 7:**
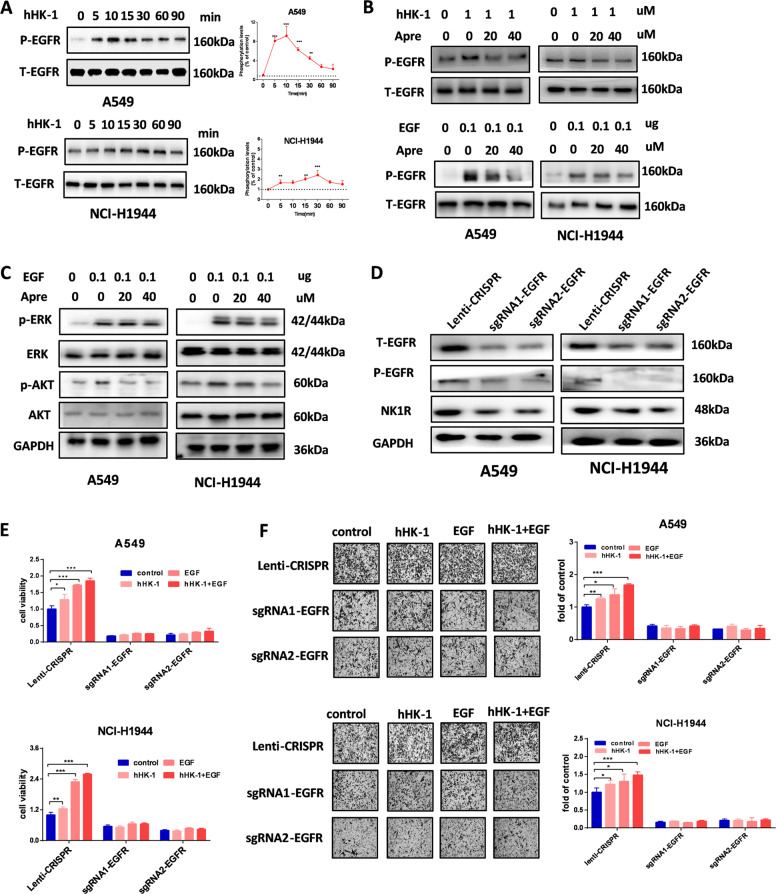
NK1R transactivates EGFR in NSCLC cells. **A** Activation of NK1R by hHK-1 transactivated phosphorylation of EGFR (pY1068). Cells were treated by 1 μM hHK-1 in indicated time after starving 2 h. **B** Aprepitant inhibited the phosphorylation of EGFR stimulated by 1 μM hHK-1 or by 0.1 μg EGF treatment. Total EGFR protein was used as loading control. **C** Aprepitant inhibited ERK1/2 and AKT phosphorylation induced by EGF. Total ERK and Akt protein were used as loading control. **D** Total or phosphorylated EGFR and NK1R expression in cells transfected with lenti-CRISPR sgRNA target to EGFR by western blot. **E** Cell proliferation in EGFR knockout cells with or without hHK-1/EGF treatment for 72 h (Celltiter-Glo assay). **F** Transwell assay for cell migration in EGFR konckout cells with or without hHK-1/EGF treatment. Data were shown as mean ± SD from three independent experiments. *P < 0.05, **P < 0.01, ***P < 0.001.

We performed confocal microscopy to determine the potential relation of NK1R and EGFR in NSCLC cells. The statistical histogram of colocalization analysis with ZEN software showed that NK1R (red) and EGFR (green) exhibited significant colocalization with an overlap coefficient >0.6 in the indicated cells (Fig. 6A and Supplementary Fig. 6A). In A549 and NCI-H1944 cells, NK1R protein was co-immunoprecipitated with EGFR and vice versus (Fig. 6B). The activation of NK1R had an influence on the interaction of NK1R-EGFR since hHK-1 augmented the interaction, while the aprepitant attenuated the effect. Moreover, when NK1R was overexpressed in NSCLC cells, the amount of co-immunoprecipitated EGFR was significantly increased (Fig. 6C). NK1R has been shown to modulate EGFR phosphorylation in breast cancer and glioblastoma cells [27, 40]. As shown in Fig. 7A and Supplementary Fig. 6B, the activation of NK1R by hHK-1 potently transactivated the phosphorylation of EGFR in a time-dependent manner in NK1R^high^ NSCLC cell lines. Selective NK1R antagonist aprepitant alleviated the phosphorylation of EGFR induced by hHK-1 (Fig. 7B). In addition, EGF-induced phosphorylation of EGFR, as well as downstream ERK1/2 and Akt, especially Akt, was also attenuated by aprepitant treatment (Fig. 7B, C). Furthermore, we performed EGFR knockdown by transfecting specific Lenti-CRISPR -V2 dual sgRNA in A549 and NCI-H1944 cells (Fig. 7D), demonstrating that both cell lines showed little responses to hHK-1 in Cell-Titre Glow and Transwell assay (Fig. 7E, F). Collectively, these data showed that NK1R interacted with EGFR and that EGFR played key role in NK1R-mediated tumor cell proliferation and migration in NSCLC.

### Combination of aprepitant with EGFR TKIs synergistically inhibited NSCLC cell proliferation

**Fig. 8:**
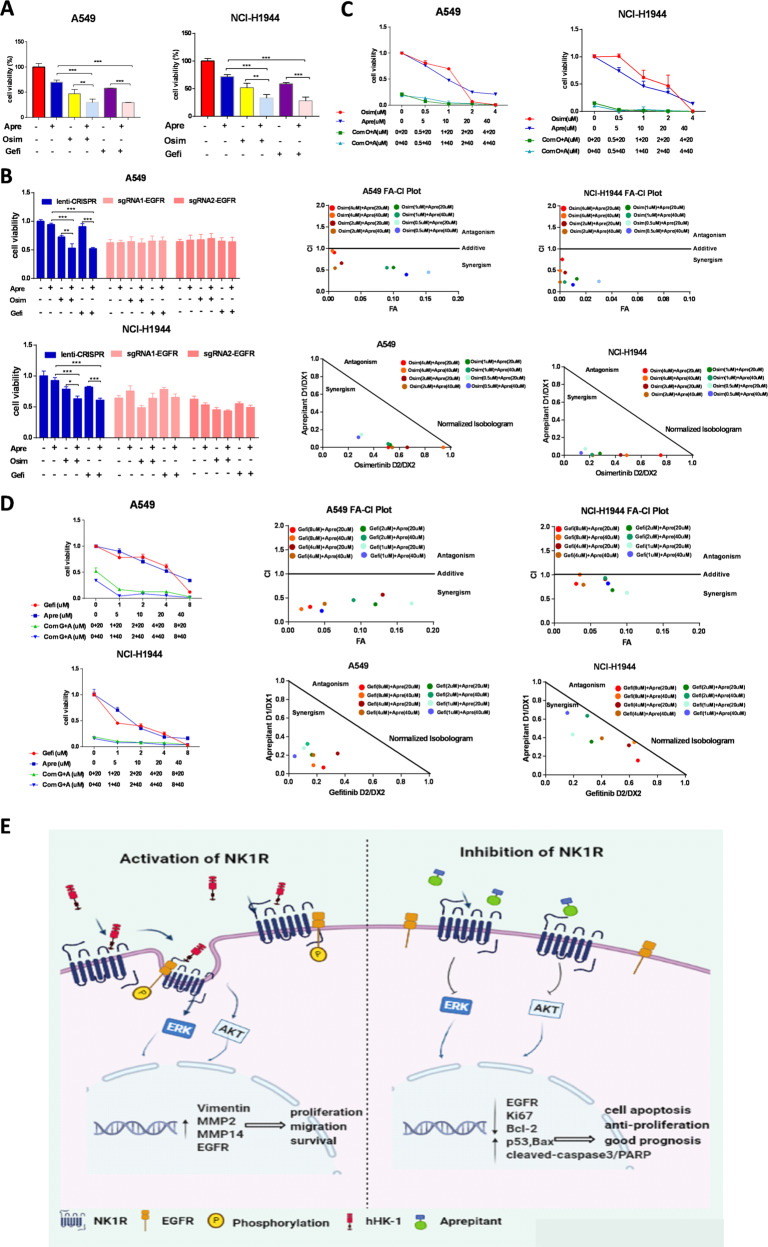
Combination of aprepitant with EGFR TKIs synergistically inhibited NSCLC cell proliferation. **A** Cell viability of A549 and NCI-H1944 treated with gefitinib (Gefi, 10 μM) or osimertinib (Osim, 1 μM) in the presence or absence of aprepitant (Apre, 20 μM). **B** Cell viability of A549 and NCI-H1944 with EGFR knockdown treated with gefitinib (Gefi, 10 μM) or osimertinib (Osim, 1 μM) in the presence or absence of aprepitant (Apre, 20 μM). **C**, **D** Cell viability of A549 and NCI-H1944 treated with Apre and Osim/Gefi at indicated concentrations. FA-CI and isobologram analysis for aprepitant/osimertinib or geftinib combinations was plotted and calculated with Chou–Talalay algorithms. CI < 1 suggest synergism effect; CI = 1 suggest additive effect; CI > 1 suggest antagonism effect. Isobologram analysis represents the synergism of a combination of aprepitant with TKIs. Data were shown as mean ± SD from three independent experiments. *P < 0.05, **P < 0.01, ***P < 0.001. **E** Working model. NK1R regulates interaction with EGFR, inducing the phosphorylation of ERK/AKT in NSCLC cells and promoting tumors progression.

NSCLC cells with wild-type or insensitive mutant EGFR are usually unresponsive to the small-molecule EGFR TKIs. Aprepitant repressed NSCLC cell proliferation (Fig. 3F). By cell viability and colony formation assay we demonstrated that aprepitant potentiated the inhibitory effect of gefitinib/osimertinib in NSCLC cells (Fig. 8A and Supplementary Fig. S7A, B); interestingly, aprepitant showed no inhibitory effect on NSCLC cells proliferation in the presence or absence of gefitinib/osimertinib when EGFR expression was knocked down, suggesting a synergistical action between NK1R and EGFR (Fig. 8B). Then we calculated the combinational effect of aprepitant with gefitinib or osimertinib by Chou–Talalay algorithms with Compusyn software. The isobologram analyses indicated that all the points were below the line of additive effects; the fraction-effect (FA) versus combination index (CI) analysis also demonstrated that aprepitant synergistically enhanced the growth inhibition induced by osimertinib or gefitinib in NCI-H1944 and A549 cells (Fig. 8C, D). Collectively, these data showed antagonist of NK1R acted in synergy with gefitinib/osimertinib to inhibit NSCLC cells proliferation.

## Discussion

Drugs targeting GPCRs have made great breakthroughs and achievements in cancer treatment [10, 41]; however, their therapeutic potentials have not been extensively evaluated in lung cancer. Here we presented evidence for the novel role of NK1R in NSCLC tumor progression through its crosstalk with EGFR. Our data revealed that NK1R was significantly upregulated in NSCLC patients’ tumor tissues compared with normal tissues and positively related to clinical classification. In stage I and II NSCLC patients, NK1R expression level was associated with poor prognosis. Notably, NK1R is co-expressed with EGFR in NSCLC patient tissues and cells. NK1R can interact and regulate EGFR signaling, inducing EGFR phosphorylation as well as an enhancement of intracellular signaling activities of ERK1/2 and Akt. Furthermore, NK1R activation can increase the proliferation, migration as well as promote EMT process in NSCLC cells. These effects are abolished by either shRNA-mediated downregulation of NK1R expression or the selective antagonist of NK1R aprepitant. Importantly, NK1R antagonist induced apoptosis in NSCLC cells and showed combinational inhibitory effect with EGFR TKIs treatments. These findings supported the remarkable role of NK1R in NSCLC development.

NK1R has been reported to be overexpressed in several types of cancer tissues, playing a substantial role in cancer cell proliferation, migration, angiogenesis, and tumor microenvironment plasticity [29]. Studies showed that NK1R regulated the growth of triple-negative breast cancer (TNBC) by transactivating EGFR phosphorylation and affected the therapeutic effect of EGFR mAb cetuximab [42]. In glioblastoma cells, EGFR was implicated to be essential in NK1R-mediated MAPK activation and cell proliferation [40]. In this study, we found that the upregulation of NK1R in NSCLC tumor tissues was positively related to the expression level of EGFR. Mechanically, activation of NK1R can transactivate EGFR by inducing EGFR phosphorylation in NSCLC cells. In consistence, as shown in Figs. 2E and 7B, C, NK1R antagonist aprepitant blocked both hHK-1- and EGF-induced EGFR, ERK1/2, and Akt phosphorylation. It is noteworthy that NK1R and EGFR protein can be co-immunoprecipitated in NSCLC cell lysates, indicating a protein–protein interaction between them (Fig. 6B, C). It is interesting that this interaction seemed to be influenced by the status of NK1R. The agonist-binding NK1R potentiated the effect, while the antagonist aprepitant attenuated it. In glioblastoma cells, NK1R-mediated EGFR transactivation was involved with a PTX-sensitive Gα protein; activated EGFR complex was found to contain adaptor proteins SHC and Grb2 [43]. Studies from our group and others have indicated that NK1R can couple with Gs or Gq protein after ligand activation. The activated NK1R can also recruit adaptor proteins like β-arrestins and c-Src [44, 45]. We speculate that the interaction between NK1R and EGFR in NSCLC cells may be involved with these adaptor proteins, although still need experimental data to validate this hypothesis.

It is observed that hHK-1 treatment increased the protein level of MMP2/14 in NSCLC cells (Fig. 2I). We proved previously that the activation of NK1R stimulated the expression and activities of MMPs like MMP2 and MMP14, involving the migration of glioblastoma, melanoma, and breast cancer cells [46–48]. In NSCLC cells, except that increased MMP2/14 could degrade the extracellular matrix and enable tumor cells to invade into the area nearby, it also provided the opportunity to transactivate EGFR. Because MMP2/14 were able to promote the shedding of EGFR ligands such as HB-EGF, TGFα, and amphiregulin, which further bind and activate EGFR signaling [49]. Indeed, bombesin receptor and endothelin receptor were shown to transactivate the phosphorylation of EGFR in NSCLC cells in the MMP-dependent way [15, 50]. NK1R-induced MMP2/14 upregulation in NSCLC cells may represent another potential mechanism underlying NK1R-mediated EGFR transactivation. Intriguingly, both western blot and real-time quantitative PCR result showed that overexpressing NK1R in NSCLC cells can further enhance the expression level of EGFR while knockdown of NK1R can reduce it (Fig. 5D, E). The study of Wang et al. also demonstrated that NK1R activation could enhance EGFR expression in TNBC cells in vitro, although they found no correlation between NK1R and EGFR expression level in TNBC cases [42]. These results implied that the crosstalk between NK1R and EGFR might also exist at the transcriptional level.

The development of NSCLC is associated with both EGFR activating mutation and wild-type EGFR amplification [51]. Our analysis showed NK1R expression level had a positive correlation with EGFR in NSCLC patient samples, but it was not clear if the upregulation of NK1R was associated with the gene status of EGFR. The expression of NK1R was detected in the NSCLC cell lines used in this study, in which A549, NCI-H1944, and NCI-H226 harbored wild-type EGFR, HCC827 harbored EGFR/Del19, and NCI-H1975 harbored EGFR/L858R/T790M. NK1R-transactivated EGFR phosphorylation and co-immunoprecipitation of NK1R and EGFR are detected in all these cell lines except HCC827, due to a relatively low expression level of NK1R in it (Figs. 6A–C and 7A and Supplementary Fig. 6A, B). These data supported that NK1R can transactivate EGFR despite its gene status. Furthermore, our results indicated that reduction of NK1R in NSCLC cell lines with either wild-type EGFR or mutant EGFR showed a significant inhibitory effect on tumor progression. In addition, NK1R antagonist aprepitant showed a combinational effect with EGFR TKI gefitinib and osimertinib in NSCLC cells. These results suggested that the upregulation of NK1R may explain the primary insensitivity or acquired resistance to anti-EGFR therapy at least in part of NSCLC patients. Recently, FDA has approved a novel anti-EGFR reagent amivantamab that is a bispecific antibody targeting both EGFR and cMet. Amivantamab treatment leads to NSCLC regression across EGFR driver mutations, including Del19, L858R, Ex20In, T790M/C797S as well as against drug resistance due to MET upregulation [52]. It deserves further effort to develop strategies co-targeting NK1R and EGFR as an alternative approach to curb tumor development in NSCLC patients driven by EGFR and/or NK1R. In a summary, the current study demonstrated the co-expression and interaction of NK1R and EGFR in NSCLC tumor cells. High NK1R expression level is associated with aggressive cancer development and poor survival probability of NSCLC patients. In conclusion, our data established a NK1R-EGFR-ERK/Akt axis to promote NSCLC tumor progression (Fig. 8E). The crosstalk between NK1R and EGFR provides a potential therapeutic target for the unmet clinical need of NSCLC patients who poorly respond to current therapeutic strategies [].

## Supplementary information

Supplementary Figure 1

Supplementary Figure 2

Supplementary Figure 3

Supplementary Figure 4

Supplementary Figure 5

Supplementary Figure 6

Supplementary Figure 7

Supplementary Figure Legends

Supplementary Table 1

Supplementary Table 2

Supplementary Table 3

Reproducibility checklist
