# Correction: Neurokinin-1 receptor promotes non-small cell lung cancer progression through transactivation of EGFR

**DOI:** 10.1038/s41419-025-07966-6

**Published:** 2025-11-20

**Authors:** Xiao-Wei Zhang, Lin Li, Wen-Qian Hu, Ming-Ning Hu, Yan Tao, Hui Hu, Xiao-Kang Miao, Wen-Le Yang, Qiong Zhu, Ling-Yun Mou

**Affiliations:** 1 School of Life Science Lanzhou University, 222 TianShui South Road, Lanzhou, 730000 P. R. China; 2 Basic Medical Sciences & Research Unit of Peptide Science, Chinese Academy of Medical Sciences, 2019RU066, Lanzhou University, Lanzhou, 730000 P. R. China; 3 Key Laboratory of Preclinical Study for New Drugs of Gansu Province, School of Basic Medical Science, Lanzhou University, Lanzhou, 730000 P. R. China; 4 Key Laboratory of Urological Disease of Gansu Province, Lanzhou University Second Hospital, Lanzhou University, Lanzhou, 730000 P. R. China

Correction to: *Cell Death and Disease* 10.1038/s41419-021-04485-y, published online 10 January 2022

We discovered that the NK1R Western-blot panel in Fig. 5E was inadvertently duplicated with Vimentin in Fig. 3E during manual layout. Our article contains 193 individual Western-blot panels distributed over 21 composite figures; the error arose while rearranging these panels in PowerPoint.

**Amended Fig 5** 
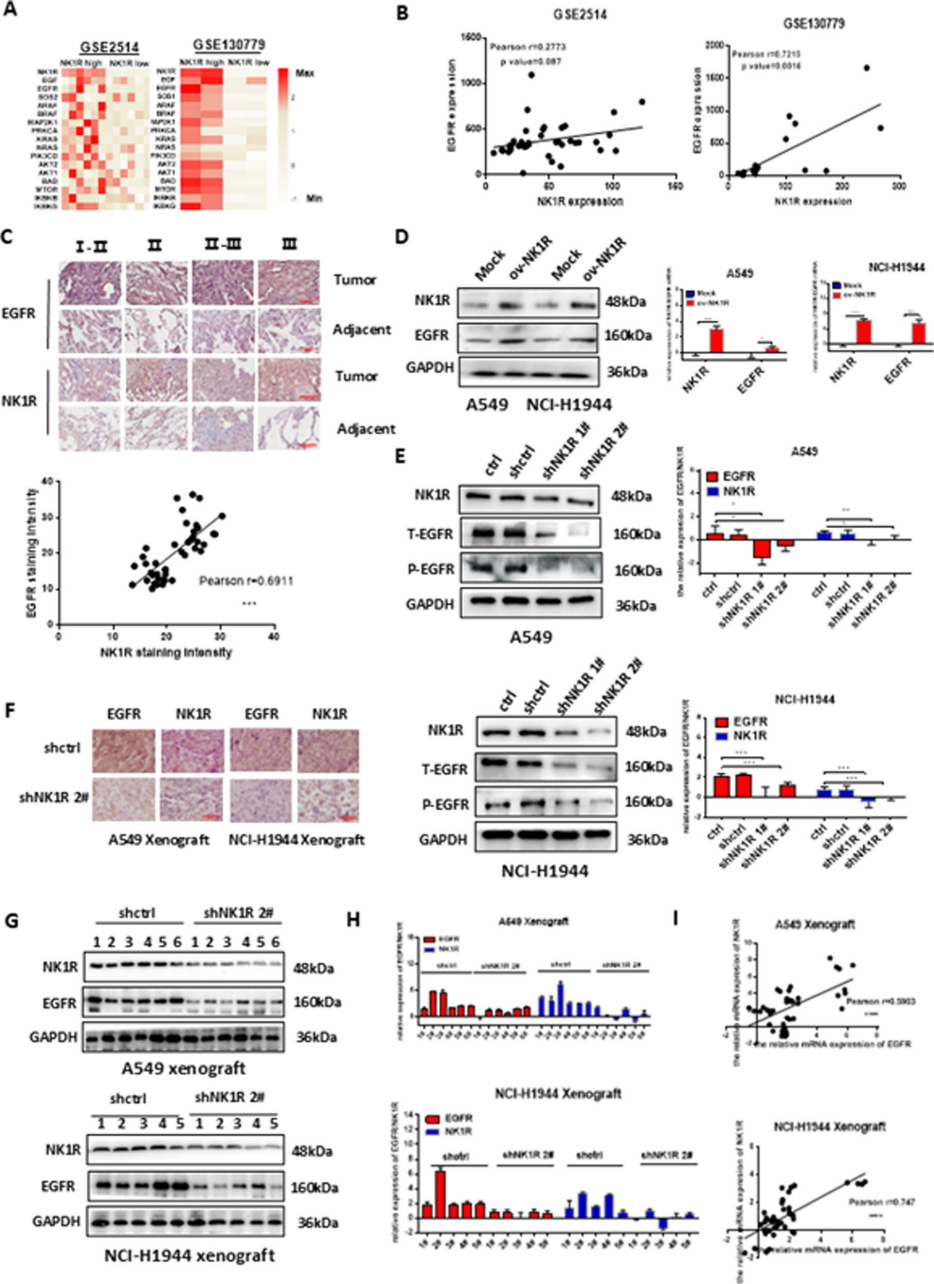


**Original data of Fig.3** 
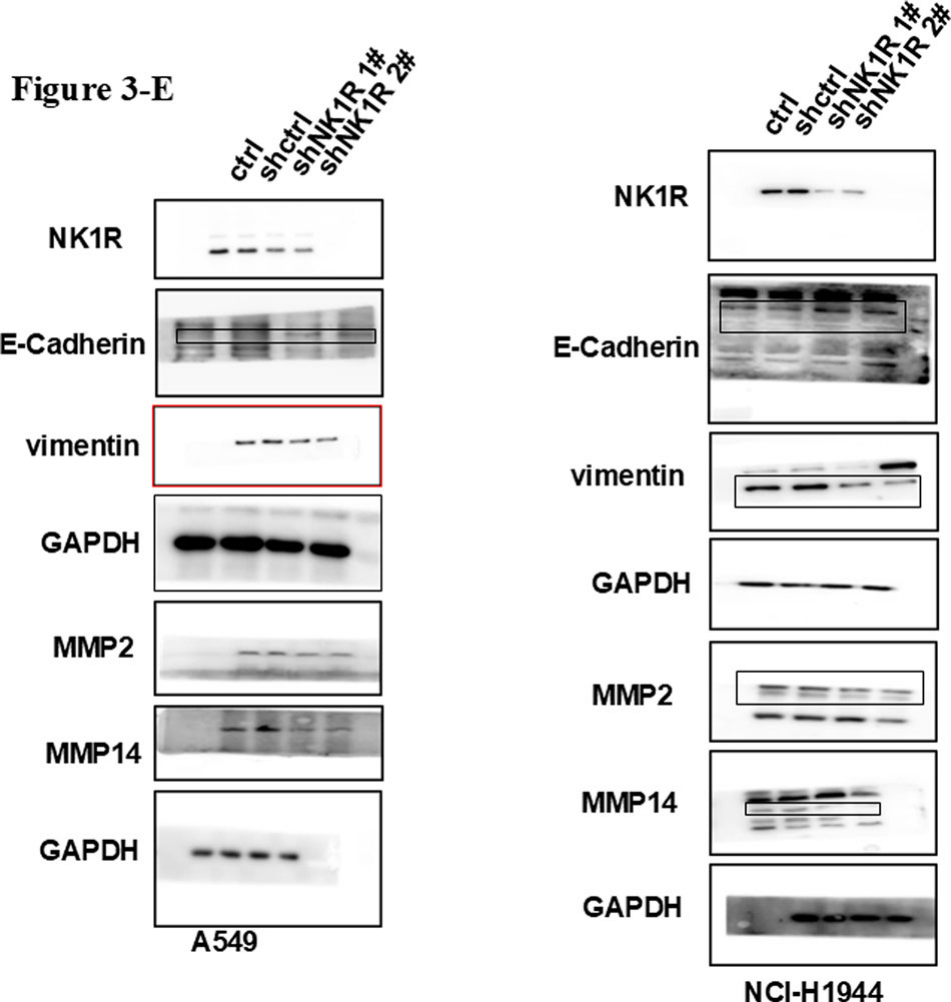


**Original data of Fig.5** 
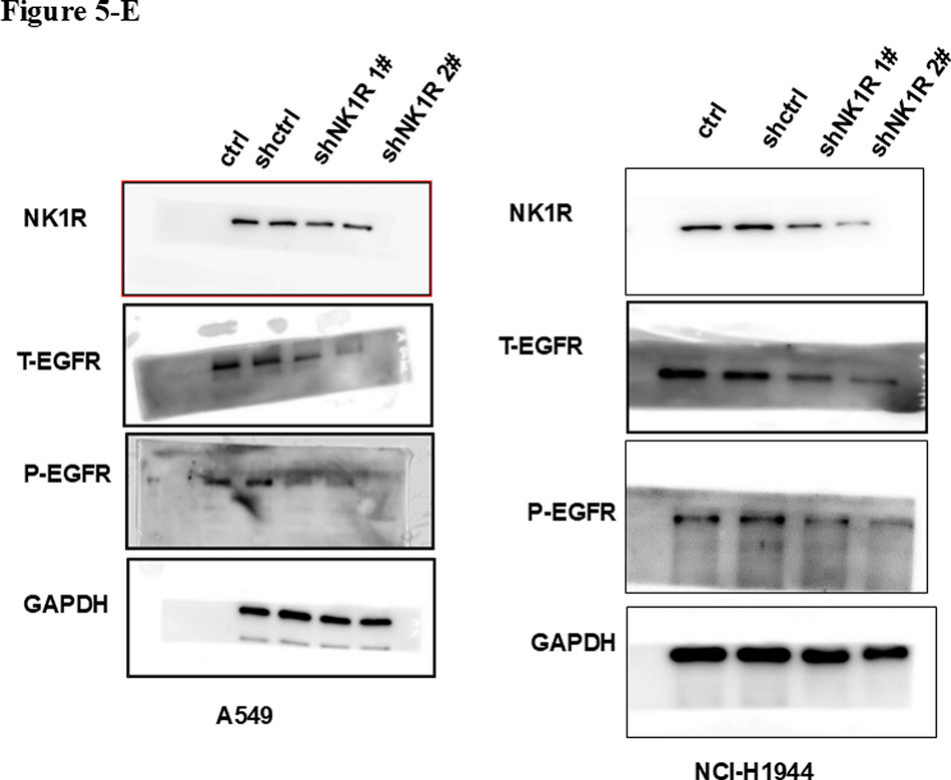


The original article has been corrected.

