# Supplementary figures and images for: Neurokinin-1 receptor promotes non-small cell lung cancer progression through transactivation of EGFR
Source: Cell Death Dis. 2022 Jan 10;13(1):41. doi: 10.1038/s41419-021-04485-y (PMC8748918; doi:10.1038/s41419-021-04485-y)

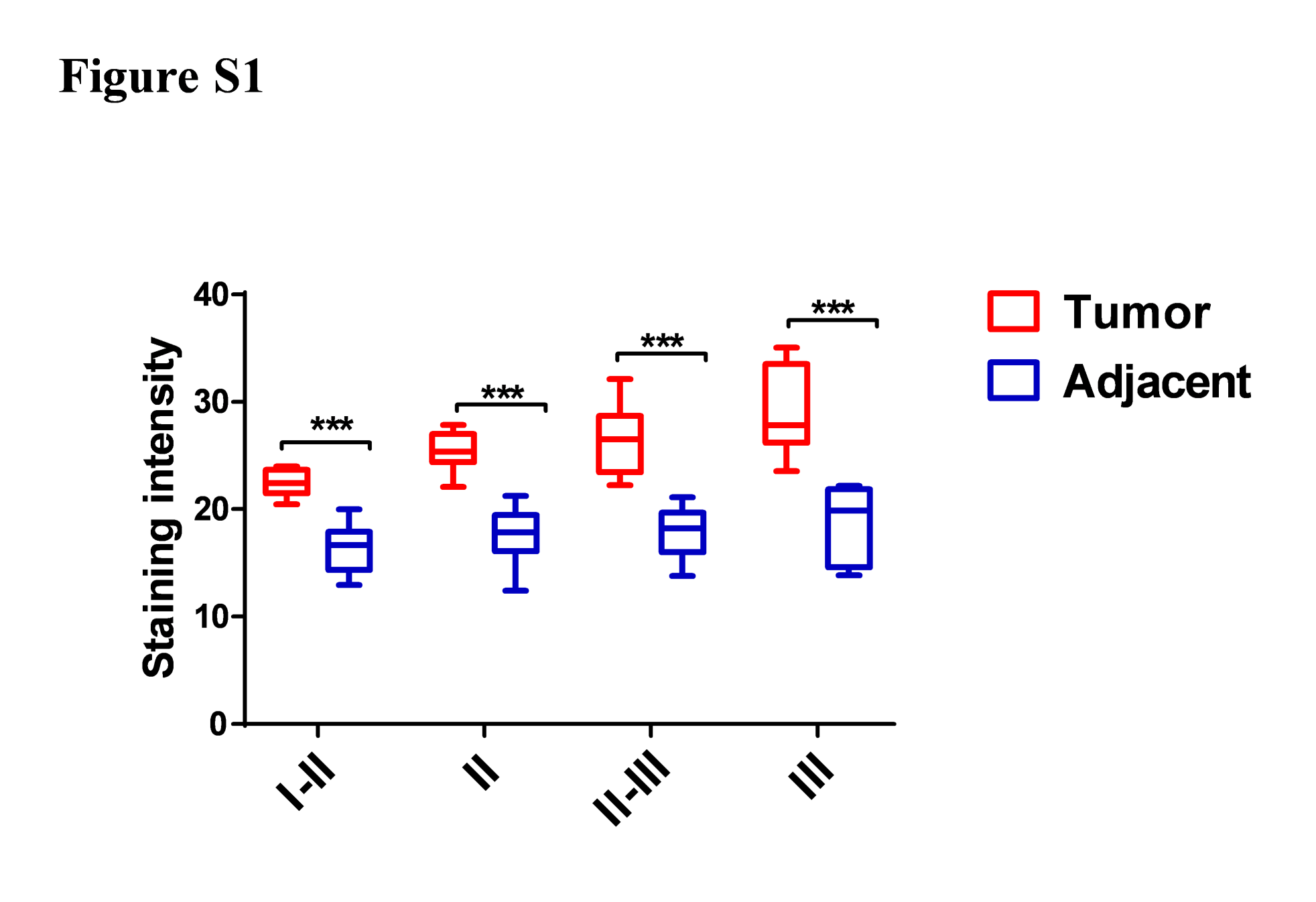

Supplement: Supplementary file 1 — Supplementary Figure 1 [file 41419_2021_4485_MOESM1_ESM.png]

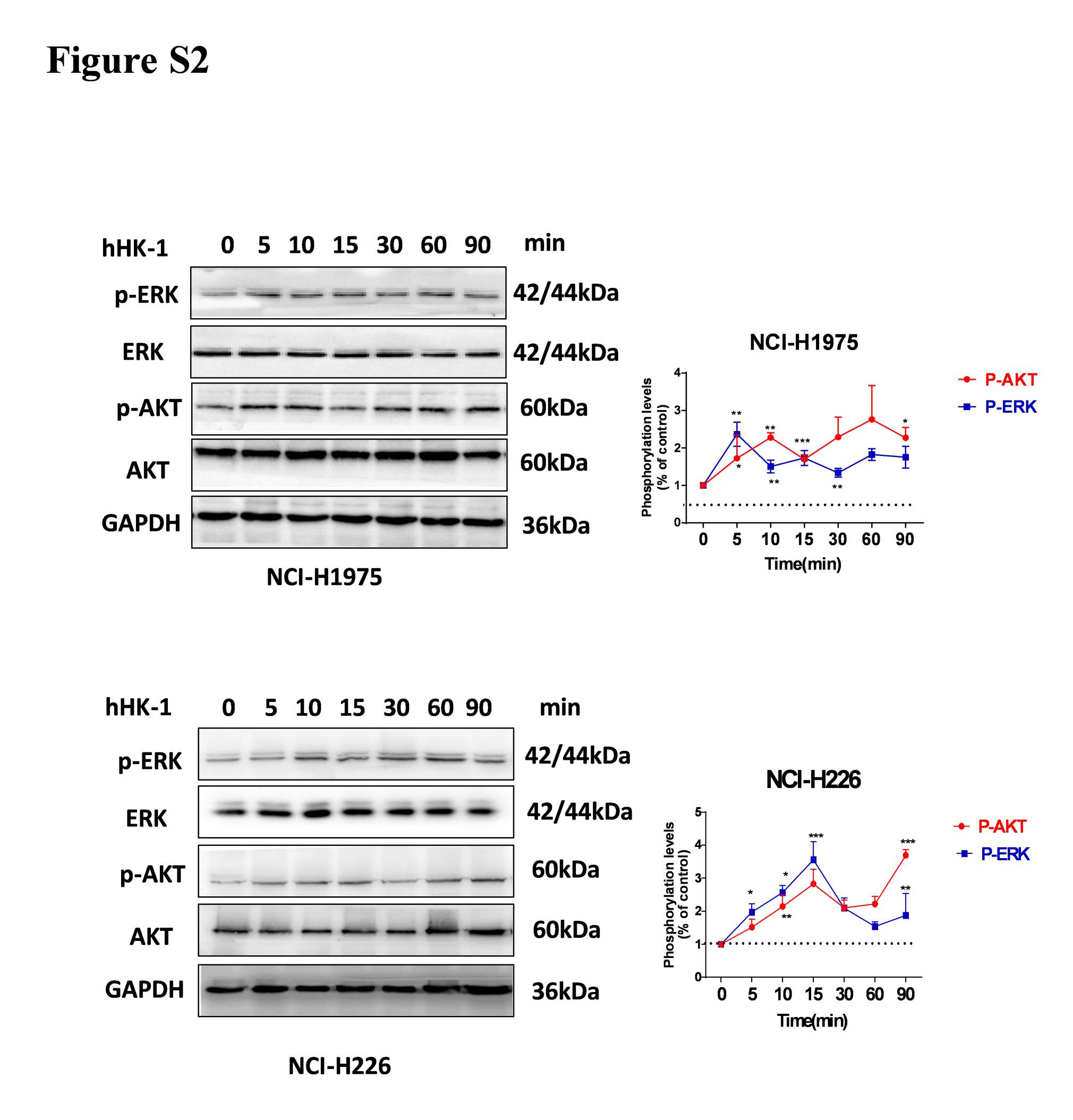

Supplement: Supplementary file 2 — Supplementary Figure 2 [file 41419_2021_4485_MOESM2_ESM.png]

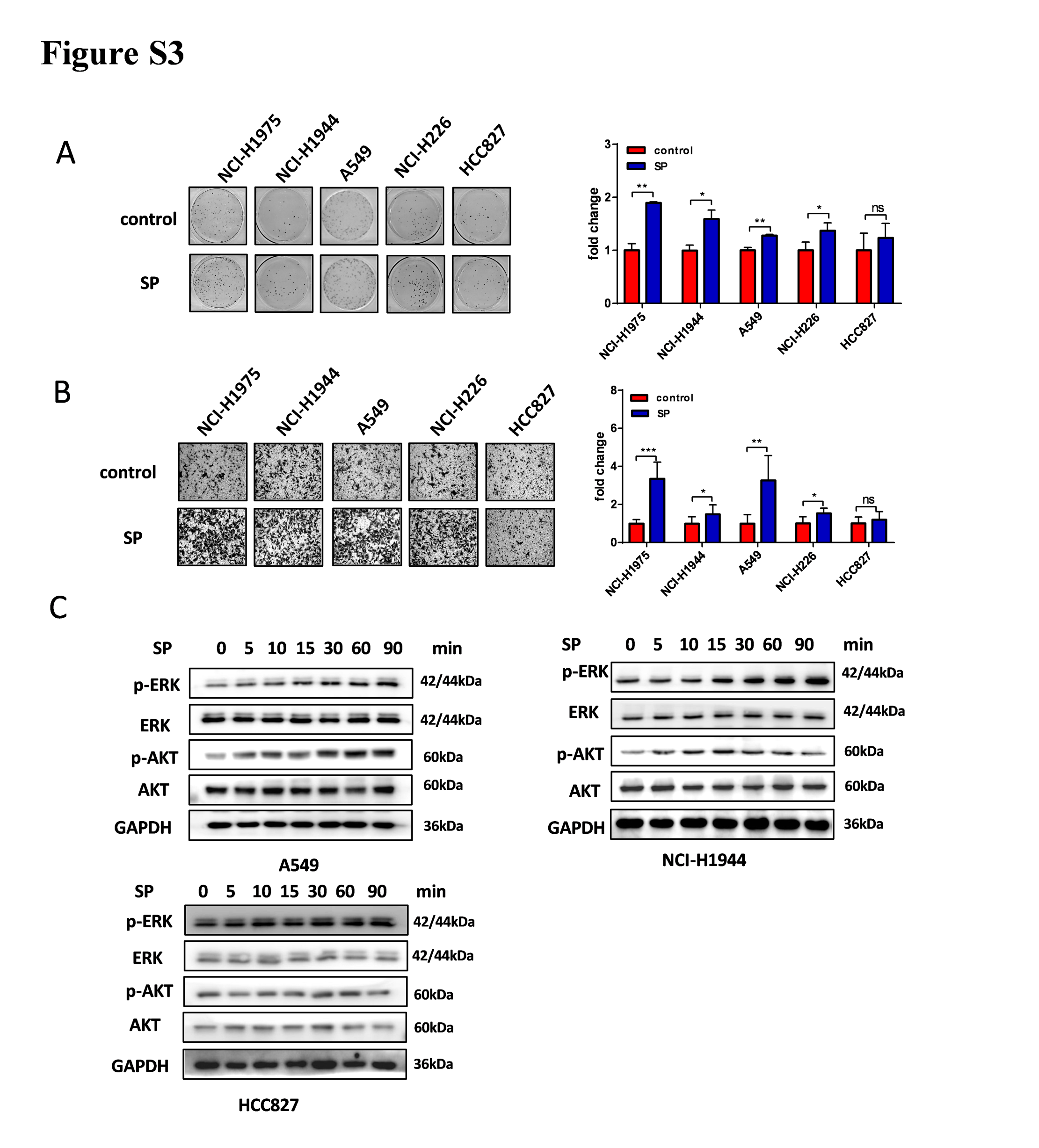

Supplement: Supplementary file 3 — Supplementary Figure 3 [file 41419_2021_4485_MOESM3_ESM.png]

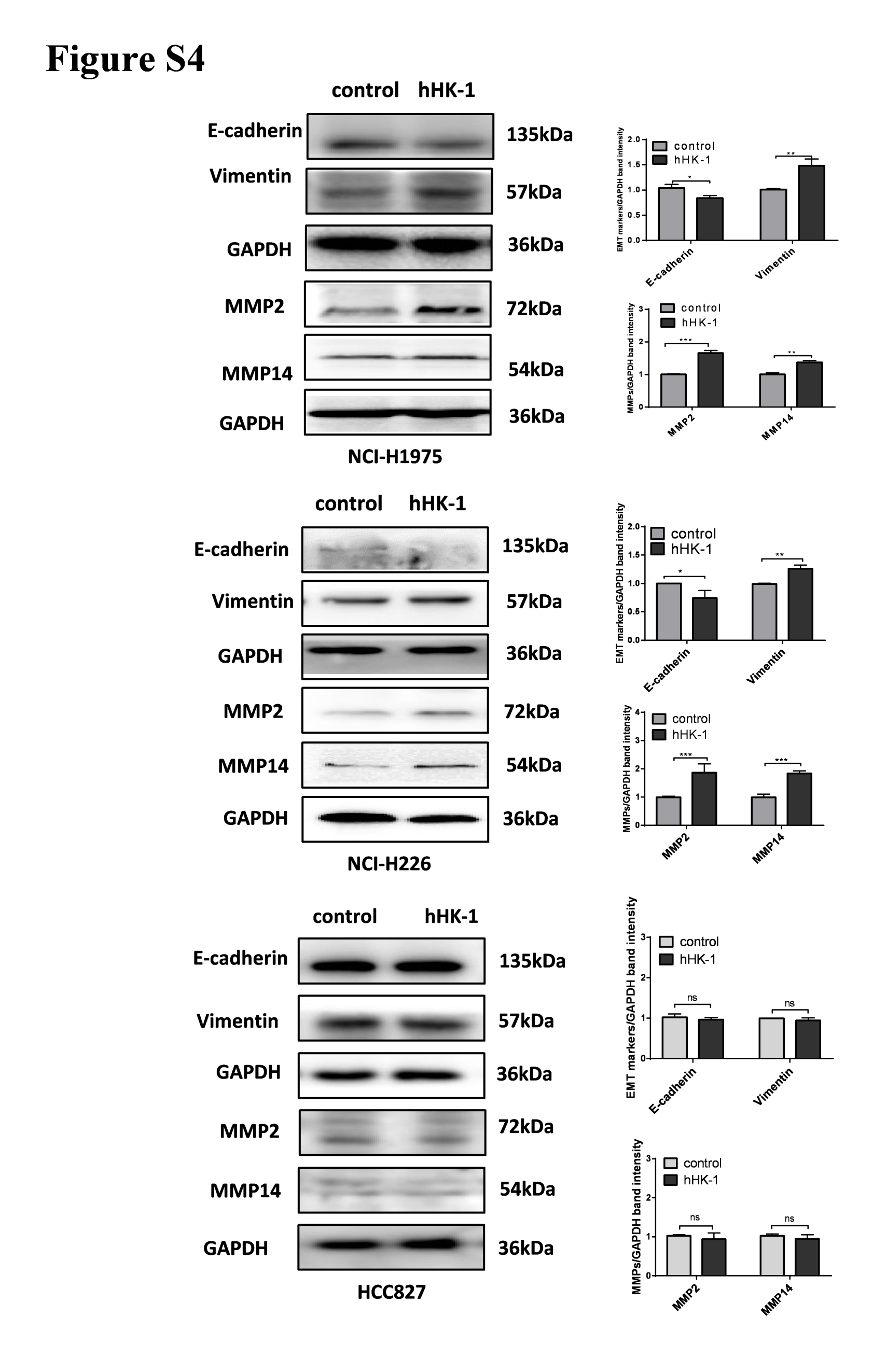

Supplement: Supplementary file 4 — Supplementary Figure 4 [file 41419_2021_4485_MOESM4_ESM.png]

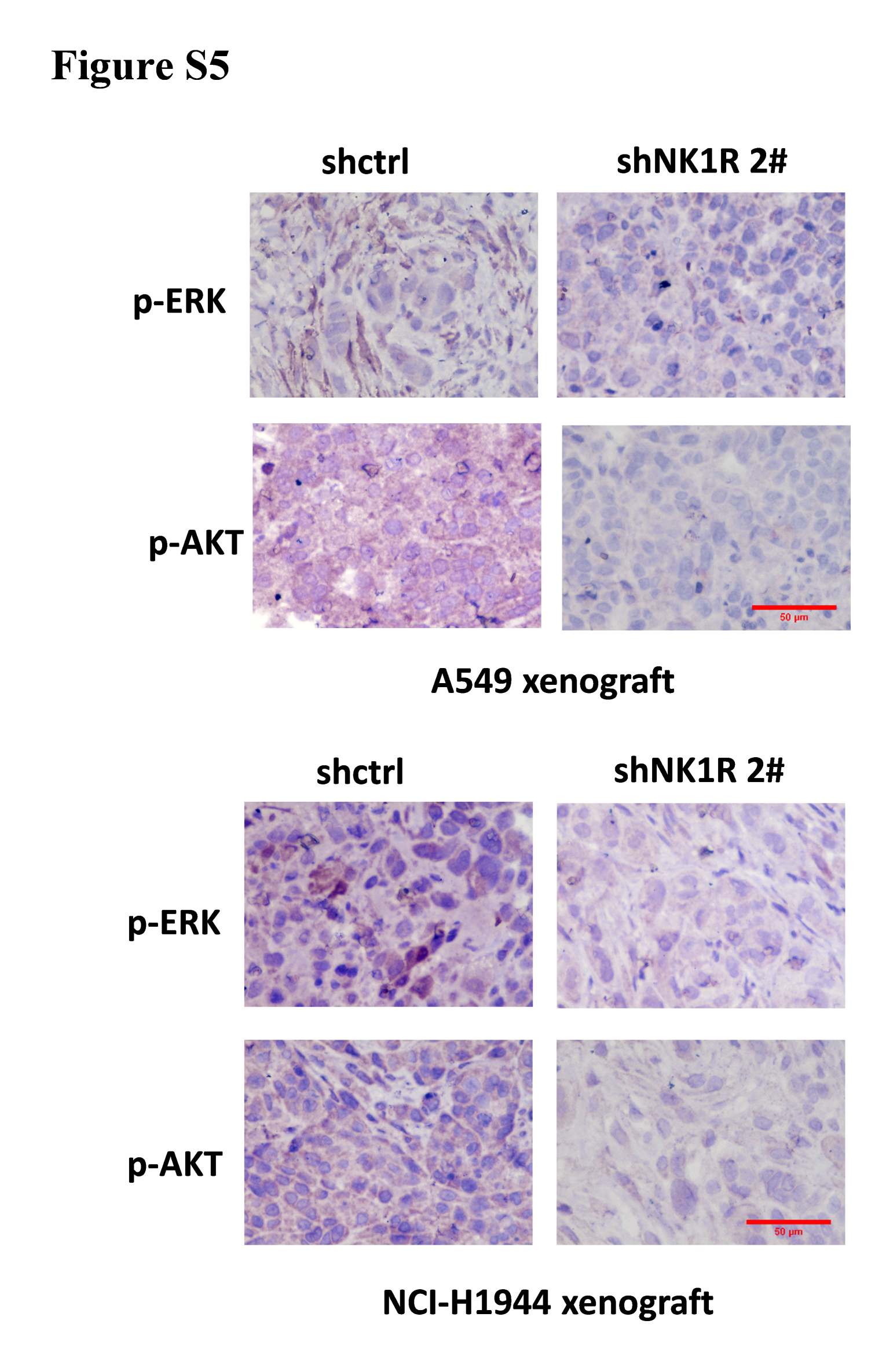

Supplement: Supplementary file 5 — Supplementary Figure 5 [file 41419_2021_4485_MOESM5_ESM.png]

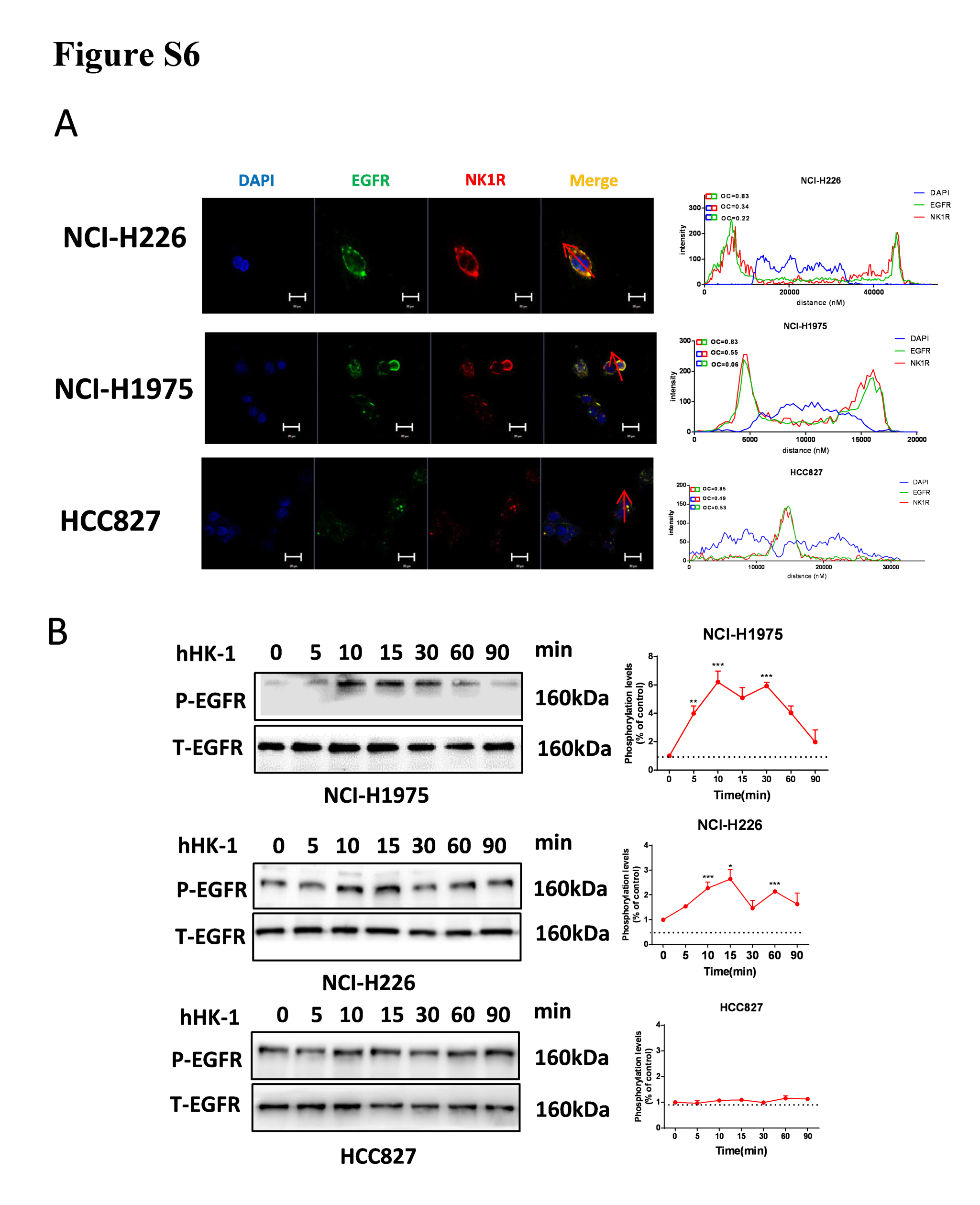

Supplement: Supplementary file 6 — Supplementary Figure 6 [file 41419_2021_4485_MOESM6_ESM.png]

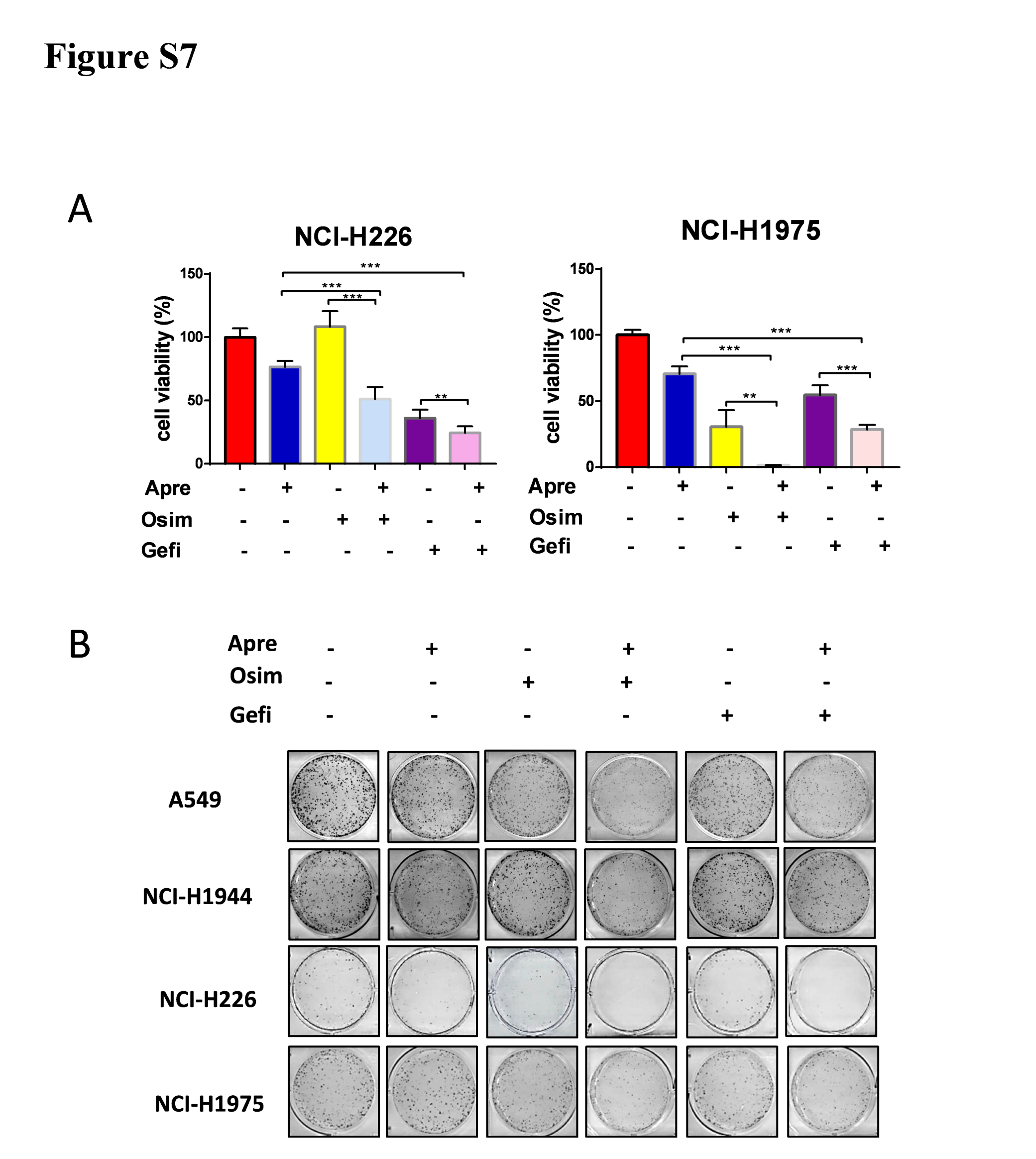

Supplement: Supplementary file 7 — Supplementary Figure 7 [file 41419_2021_4485_MOESM7_ESM.png]
