# Supplementary material for: Neurokinin-1 receptor promotes non-small cell lung cancer progression through transactivation of EGFR
Source: Cell Death Dis. 2022 Jan 10;13(1):41. doi: 10.1038/s41419-021-04485-y (PMC8748918; doi:10.1038/s41419-021-04485-y)
Supplement: Supplementary file 8 — Supplementary Figure Legends [file 41419_2021_4485_MOESM8_ESM.docx]

**Supplementary Figure Legends**

**Figure S1.** Immunohistochemistry (IHC) staining score of NK1R expression in lung cancer tissue and adjacent non-tumor was calculated in different clinical grade from 30 paired lung cancer tissues by Image-pro Plus 6.0. Error bar was signified mean ± SD from repeat independent experiment. P*<0.05, P**<0.01, P***<0.001.

**Figure S2.** The phosphorylation level of ERK1/2 and AKT was detected in cells pretreated with starving 2 h with 1uM hHK-1 treatment by western-blot. Band intensity was analyzed by Image J, and GAPDH was used the normalized protein. Data was shown as mean ± SEM from at least 3 repeated and independent experiments. P*<0.05, P**<0.01, P***<0.001.

**Figure S3. (A)** Colony formation assay and **(B)** transwell assay were performed in NSCLC cells for NK1R function under SP stimulation in cell growth and migration. **(C)** The cells pretreated with starving 2 h and stimulated 1uM hHK-1 by western-blot for the phosphorylation level of ERK1/2 and AKT. Data was shown as mean ± SEM from at least 3 repeated and independent experiments. P*<0.05, P**<0.01, P***<0.001.

**Figure S4.** E-cadherin, vimentin and MMP2/14 in NSCLC cells with hHK-1 treatment for 24 hours in NCI-H1975, NCI-H226 and HCC827 cells by western blot. Band intensity was analyzed by Image J, and GAPDH was used the normalized protein. Data was shown as mean ± SEM from at least 3 repeated and independent experiments. P*<0.05, P**<0.01, P***<0.001.

**Figure S5.** The phosphorylation level of ERK1/2 and AKT was detected in in NK1R-knockdown tumor xenograft tissues by IHC staining (magnification, x40).

**Figure S6. (A)** Confocal microscopy analysis to detect the colocalization of EGFR and NK1R in NCI-H226, NCI-H1975 and HCC827. Green, EGFR; Red, NK1R; nucleus was stained by DAPI. The plot profile of fluorescence intensity was used to show the overlap of the NK1R and EGFR in the same location. The value of Overlap Coefficient (OC) was > 0.6 as the indication co-localization, while overlap coefficient was < 0.6 as the absence of co-localization. **(B)** Activation of NK1R by hHK-1 transactivated phosphorylation of EGFR (pY1068). Cells were treated by 1 μM hHK-1 in indicated time after starving 2h. GAPDH was used the normalized protein. Band intensity was analyzed by Image J. Data was shown as mean ± SEM from at least 3 repeated and independent experiments. P*<0.05, P**<0.01, P***<0.001.

**Figure S7. (A)** Cell-titer Glo assay and **(B)** Colony formation of NSCLC cell viability. Cells were treated with gefitinib (Gefi, 10 μM) or osimertinib (Osim, 1 μM) in presence or absence of aprepitant (Apre, 20 μM) at indicated concentration. Data was shown as mean ± SEM from at least 3 repeated and independent experiments. P*<0.05, P**<0.01, P***<0.001.
